# The carboxyl terminal trimer of procollagen I induces pro-metastatic changes and vascularization in breast cancer cells xenografts

**DOI:** 10.1186/1471-2407-9-59

**Published:** 2009-02-18

**Authors:** Davide Visigalli, Daniela Palmieri, Antonella Strangio, Simonetta Astigiano, Ottavia Barbieri, Gianluigi Casartelli, Antonio Zicca, Paola Manduca

**Affiliations:** 1 Laboratorio di Genetica, Dip. Biologia, Università di Genova, Italy; 2 DIMES, Università di Genova, Italy; 3 Istituto Nazionale per la Ricerca sul Cancro, Genova, Italy

## Abstract

**Background:**

The COOH terminal peptide of Pro-collagen type I (PICP, also called C3) is chemotactic for endothelial melanoma and breast cancer cells. PICP induces the expression of Metalloproteinases-2 and -9, of Vascular endothelial growth factor and of the chemokine CXCL-12 receptor CXCR4 in MDA MB231 breast carcinoma cells in vitro.

**Methods:**

We used a model of xenografts in BalbC/nude mice obtaining tumors by implanting in contro-lateral subcutaneous positions MDA MB231 cells added or not with purified PICP and studied the earlier phases of tumor development, up to 48 days from implant, by histology, immunostain and in situ hybridization.

**Results:**

Addition of PICP promotes rapid vascularization of the tumors while does not affect mitotic and apoptotic indexes and overall tumor growth. PICP-treated, relative to control tumors, show up-modulation of Vascular endothelial factor, Metalloproteinase-9 and CXCR4, all tumor prognostic genes; they also show down-modulation of the endogenous Metalloproteinase inhibitor, reversion-inducing-cysteine-rich protein with kazal motifs, and a different pattern of modulation of Tissue Inhibitor of Metalloproteinase-2. These changes occur in absence of detectable expression of CXCL-12, up to 38 days, in control and treated tumors.

**Conclusion:**

PICP has an early promoting effect in the acquisition by the tumors of prometastatic phenotype. PICP may be play a relevant role in the productive interactions between stroma and tumor cells by predisposing the tumor cells to respond to the proliferation stimuli ensuing the activation of signaling by engagement of CXCR4 by cytokines and by fostering their extravasion, due to the induction of increased vascular development.

## Background

Peritumoral stroma is involved in the development of tumors, induces changes in gene expression conductive to cancer progression and promotes inflammation, angiogenesis and metastasis [1-6]. In breast carcinomas changes occur in the expression of genes involving all tumor-infiltrating cell types [7]. In breast cancer, stromal fibroblasts respond to pre-tumoral cells by undergoing epigenetic changes, which lead to altered molecular composition of the extracellular matrix (ECM) [8,9], with increased deposition of collagens, fibronectin and tenascin [10], and increase in inflammatory cytokines and growth factors, such as VEGF and CXCL-12, which promote vascular development and cancer cell proliferation and invasiveness [11-15].

Vascular endothelial Growth factor (VEGF)-A is a major angiogenic factor. The activation of an autocrine loop of VEGF-A signaling required for breast carcinoma invasion in vitro, is mediated by the receptor Neuropilin-1(Nrp-1), but not the receptor Flt-1 [15]. Down regulation of the inhibitory forms of VEGF-A (VEGF-xxxb), due to differential splicing at the C terminus, is accompanied by up-modulation of total VEGF-A and is associated with tumor progression and angiogenesis [16].

Autocrine VEGF regulates the expression of the chemokine receptor CXCR4, which mediates the migration of breast carcinoma cells toward CXCL-12, required for tumor cells invasion but not for their survival [17]. The activation of a paracrine loop CXCR4/CXCL-12 involving tumor cells and stromal fibroblasts induces tumor growth in implants of MCF-7 breast carcinoma cells with cancer associated fibroblasts (CAF) [14]. Activation of CXCR4/CXCL-12 signaling plays a critical role in growth and invasion of primary and metastatic breast tumors [14,18-21]; CXCR-4 expression is a hallmark of a subset of stem breast cancer cells, with metastatic behavior and homing to bone.

Cancer development also involves ECM degradation by proteases, involved in the migration of tumor cells and leading to the release of growth factors and cytokines stored in the matrix and active in regulating the survival, growth and invasiveness of cancer cells and in angiogenesis. Several proteases are implied in oncogenesis; among them members of the family of Metalloproteinases (MMP) are up modulated in neoplastic stroma [21-24]; In particular, over expression of MMP-9 is associated with malignancy and poor prognosis in breast cancer [25].

The metastatic phenotype implies multiple changes; in particular co-expression of CXCR4, VEGF-A and MMP-9 is strongly correlated with lymph node metastasis and is a prognostic marker of the metastatic ability of primary breast cancer [26].

The membrane-anchored MMP-regulator reversion-inducing-cysteine-rich protein with kazal motifs (RECK) regulates tumor invasiveness and, via direct interaction, negatively regulates MMP-9 and inhibits tumor invasion and metastasis [27,28]. RECK down regulation in solid tumors correlates with poor prognosis, while its forced expression in tumor cells results in suppression of angiogenesis, invasion, and metastasis and restoring expression of RECK in malignant cells results in suppression of invasive activity and decrease in MMP-9 secretion [29]. RECK also inhibits MMP-2 and MT1-MMP, also involved in cancer progression. Vascular sprouting is suppressed in ectopic tumors derived from RECK-expressing fibrosarcoma cells, implicating RECK also in tumor angiogenesis [30].

Tissue inhibitor of Metalloproteinases (TIMP)-2 is a stechiometric cofactor in MT1-MMP-dependent MMP-2 activation and, in excess, it acts as inhibitor of MMP-2 [31]. In prostate cancer TIMP2 and MMP2 over expression was significantly associated with a shorter disease free survival [32].

The COOH terminal peptide of Pro-collagen type I (PICP) is a stromal component produced by the proteolytic cleavage of pro-collagen I by Bone Morphogenetic Protein I during the physiological processing of collagen fibers. PICP is abundant in tissues with high expression of collagen I such as bone and the stroma of breast cancer and high levels of PICP are found in the sera of breast cancer patients and are associated with poor prognosis [33,34]. PICP induces directional migration and increased expression of MMP-2 and -9 in various breast carcinoma and melanoma cell lines and chemo attracts endothelial cells [35-37]. In MDA MD231 cells, PICP induces the expression of MT-1 MMP, MMP-2 and -9 and TIMP-2 and, in a timely succession, that of VEGF-A, -B and Nrp-1 mRNAs and VEGF-A protein, mediated by activation of p-38, and of CXCR4 mRNA and protein [35,36]. Furthermore, PICP is chemoattractant for endothelial cells in vitro [37]. These data lead us to hypothesize that PICP induces epigenetic changes in breast tumor cells, resulting in promotion of tumor vascularization and progression *in vivo*.

We have observed in BalbC/nude mice that subcutaneous inoculations of MDA MB231 cells treated with PICP results in tumors that are more vascularized than untreated, contro-lateral, tumors. Pro-metastatic genes (VEGF-A, MMP-9 and CXCR4) are up-regulated, and the tumor inhibitory gene (RECK) is down-regulated in xenograft tumors developing in presence of PICP. Growth, apoptotic and mitotic indexes of tumors are unaffected by PICP treatment. The results point to an epigenetic role for PICP in promoting breast tumor progression through the induction of the expression of genes associated to metastasis and vascularization.

## Methods

### Cell cultures and preparation of PICP

The breast carcinoma MDA MB231 cell line was cultured in DMEM 10% FCS. PICP was purified from serum free medium conditioned by mature rat osteoblasts as previously described (37). The MDA MB231 cell line expresses low endogenous levels of CXCR4, stimulated by PICP in vitro and does not express CXCL-12 in presence or absence of PICP.

### Xenografts

Six-to-eight week old females BalbC/nude mice (Charles River, Italy) were housed at 12-hour light/dark cycle at 22°C and fed ad libitum. Animals were treated according to the standards approved by the Ethical committee of the Istituto Nazionale per la Ricerca sul Cancro (National Institute or Cancer Research, IST). Mice were injected subcutaneously with 1 × 10^6 ^MDA-MB231 cells embedded in 150 μl of Growth Factors-free matrigel (Becton Dickinson, USA) in PBS added or not with PICP. Injections were performed bilaterally in the dorsal side, each animal receiving on one side cells added with PICP and on the other cells in PBS. Thereafter, mice were injected weekly with 30 μl of either PBS or PICP at the site of the original implant. All injected mice developed tumors. Tumors size was evaluated three times per week with a caliper. Mice were sacrificed after 7, 14, 21 or 28 days from implantation in a set of experiments and or after 24, 31,38,48 in another. At each time point six to ten animals were sacrificed. Tumors were dissected, weighted and fixed overnight in 4% paraformaldehyde at 4°C, washed extensively in PBS, saturated with increasing concentrations of sucrose and embedded in O.C.T. (Tissue-Tek) for preservation at -80°C or in paraffin. Serial sections 6 μm thick from criopreserved tumors were used for morphology (hematoxylin-eosin stain), mitotic and apoptotic indexes determination (by Giemsa and Hoechst stains) and CD31 detection by immunohistochemistry and immunofluorescence. Paraffin embedded tumors were sectioned in 4 μm tick slices and used for in situ hybridization and all other immunohistochemistry. Images were captured with the Olympus Camedia Digital camera C3030 attached to an Olympus BX51 Microscope (Japan).

### Mitotic and apoptotic indexes

Criopreserved tumors were sectioned and slices were treated either with Giemsa stain (3% in Sorensen's buffer, pH 6.8, 30') or Hoechst 33258 stain (0.2 mM in phopsphate-buffered saline PBS 40' in the dark) at room temperature. These last slides were mounted with Vectashield and cells were scored using a UG1 excitation filter and a combination of dichroid mirrors and secondary filters transmitting light at 460–500 nm. Scoring was done an Axioplan Zeiss fluorescence microscope (Germany) equipped with a two-wavelength filter combination. For each slide a minimum of 1,000 cells were scored in randomly chosen fields.

### Immunohistochemistry and immunofluorescence

Tumor frozen sections were fixed 10 minutes in cold methanol:acetone (1:1), blocked in PBS-10% goat serum and incubated with a rat monoclonal anti-CD31 (from Dr. Laura Borsi, Italy). Secondary antibodies were anti-rat either conjugated with TRITC (Sigma), or with biotin (BioSpa) followed by alkaline phosphatase-conjugated streptavidin (BioSpa) and developed with Fast Red (LabVision) in the presence of Levamisole.

From tumors embedded in paraffin slices were processed for immunoperoxidase after citrate unmasking of the antigen at 95°C, peroxidase quenching, rinses in PBS and incubation with primary antibody for 60' at room temperature. Incubation for 60' with the secondary antibody (HRP polymer conjugate broad spectrum DAB kit from Zymed Laboratories inc., cat 87-9663), was followed by Haematoxylin/Eosin staining. Controls were without primary antibody. Primary antibodies were used at 1/50 Dilution for Ab-110 to MMP-9 polyclonal in rabbit (donated by Dr. W.G. Stetler-Stevenson, CCR, NCI, NIH, Bethesda, MD, USA), Ab to VEGF-A polyclonal in rabbit (sc-507, Santa Cruz, USA, recognizing aa 1–140 of VEGF-A, and thus all forms of VEGF-A), Mab to TIMP-2 monoclonal in mouse (MAB13446, Chemicon, USA) and Ab to CXCL-12 rabbit polyclonal (sc-28876, Santa Cruz). Dilution were 1/30 for Ab to RECK polyclonal in rabbit (sc-28918, Santa Cruz) and 1/20 for monoclonal antibody in mouse to CXCR4 (MAB 170, R&D, USA) and for monoclonal antibody in rabbit to CD34 (sc-18917, Santa Cruz, USA).

### In situ hybridization

Probes were: VEGF-A, 700 bp cDNA (recognizing all VEGF-A mRNA splicing variants) cloned by RT-PCR with the PCR TA cloning kit (Invitrogen, USA) from human total RNA (primers VEGF-A human: Lp CCTTGCTGCTCTACCTCCAC Rp TCTGTCGATGGTGATGGTGT) in PCR 2.1 vector. CXCR4, 692 bp (recognizing two mRNA splicing variants) cloned by RT-PCR with PCR-Script Amp cloning kit (Stratagene, USA) from human total RNA (primer CXCR4 human Lp ATGCAAGGCAGTCCATGTCAT Rp TCTGTCGATGGTGATGGTGT) in pPCR-script amp SK(+) vector.

MMP-9, 1067 bp human cDNA cloned in Puc19 kindly provided by Prof. W.G. Stetler-Stevenson. Linearized plasmids were used to obtain sense or antisense digoxigenin-labeled riboprobes with dig-labeling nucleic acids kit (Boeringher-Mannheim, Germany) from the appropriate promoter. Ish reactions were performed on serial sections of 4 μm thick, deparaffined, dehydrated and post-fixed with 4% PFA pH 9.5 in PBS 1× [39]. After digestion with HCl 0.2 M and Proteinase K 10 μg/ml at 37°C, hybridization was performed in 3× SSC, 1 mg/ml tRNA, 10 mM DTT, Denhardt's solution 1×, 50% formamide, 1 mg/ml denatured salmon sperm, 10% dextran sulfate and 1 ng/μl of the denaturated probe, maintained overnight at high stringency [40]. Temperatures of annealing and stringency of washes were optimized for each probe. The slides were washed in 2× SSC, digested with RNase A 100 μg/ml at 37°C degrees, incubated overnight at 4 degrees with anti-digoxigenin Fab fragments conjugated to alkaline phosphatase (Boeringher-Mannheim, Germany) in buffer with 1.5 M NaCl, 0.1 M TRIS-HCl, 2 mM MgCl_2_, 0.3% Triton X-100, 10% fetal calf serum, pH 7.5. Color development was in buffer 0.1 M NaCl, 0.1 M TRIZMA base, 5 mM MgCl_2_, 10% polyvinyl alcohol 89–98 kDa (Sigma, USA), 1 mM levamisole (Sigma, USA), 0.16 mg/ml BCIP, 0.33 mg/ml NBT (Roche, Germany), pH 9.5, for 3–6 hours. Slides were counter stained with nuclear fast red 0.005% and mounted in glycerol gelatin (Sigma, USA). Here are shown exemplary micrographs. The images were captured with a Leica DMBR microscope mounted with a Leica camera DFC320 and acquired with Leica Firecam version 1.9.1. For each probe at least two different tumors per time point were analyzed and gave consisting results.

### Statistics

For mitotic and apoptotic indexes standard deviations are shown. For tumor weight one way ANOVA was utilized to define significance.

## Results and discussion

We chose an ectopic model in BalbC/nude mice for the generation of tumors by subcutaneous implantation of MDA MB231 cells, either treated or not with PICP and injected in contro-lateral locations in each mouse, in order to decrease the experimental variation due to individual differences.

**Figure 1:**
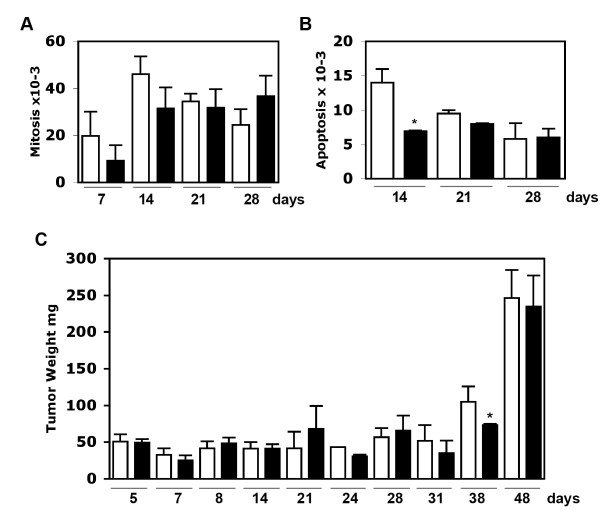
**Mitotic and apoptotic indexes and tumor weight**. A) Mitosis. B) Apoptosis. Slides were prepared from criopreserved tumors. Respectively, were analyzed slides from tumors for each condition derived from 3 mice at 7 days, 8 mice at 14 days, 6 mice at 21 days and 9 mice at 28 days. Slides were stained with Giemsa and with Hoechst stain. The results were similar for either stain and here are presented the data from Giemsa stained slides. For each slide at least 1000 cells were screened in randomly chosen fields, for a total of cells varying from 3000 to 9000 per point in each condition. Bars indicate S.D. C) Weight in mg of dissected tumors. 3 tumors for each condition were analyzed at 7, 24, 31, 38 and 48 days, and from 6 to 9 tumors for all other time points in each condition. Bars indicate S.D and the asterisks show the significance by one-way ANOVA. White columns are PBS-mock treatment controls and black columns are PICP-treated tumors.

In both control and PICP-treated xenograft tumors, no differences were observed in mitotic indexes at any of the experimental times (Fig. 1A). The only exception was for PICP-treated tumors at 14 days (Fig. 1B). As shown in Fig. 1C this last difference did not affect significantly the tumor growth: the overall growth of the tumors was similar in both control and PICP-treated tumors, with the only significant difference (p = 0.0181) found at 38 days. This difference disappeared by 48 days and it could be due to a short delay in the start of the more rapid proliferation phase that all tumors anyway undertake between 38 and 48 days. Overall, the measures of mitotic and apoptotic frequencies and of weight indicated lack of any continued effect of PICP on tumor cell proliferation within the 48 days of the experiments.

**Figure 2:**
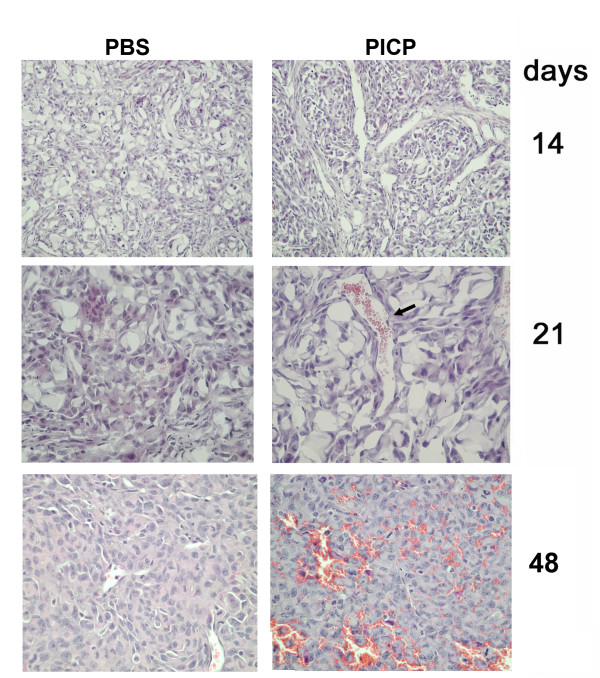
**Morphology of subcutaneous tumors in xenografts of MDA MB231 in presence or absence of PICP**. Histology of subcutaneous tumors generated by MDA MB231 and injected weekly with either vehicle or with PICP. Slices of criopreserved tumors collected after 14, 21 days and of paraffin embedded tumors at 48 days were stained with hematoxylin and eosin. Micrographs from individual tumors shown here are representative of the findings in 3 to 8 tumors for each time point. Note the presence of large vessels containing blood cells in the PICP-treated tumors, not observed in the control tumors, at 21 days and the diffuse presence of vessels at 48 days in C3 treated tumors. Enlargement is: first row 50×, second and third row 100×.

PICP treatment had an early and continuing effect in promoting vessels formation and development. By hematoxylin-eosin staining, in all the PICP-treated tumors more vessels were detected since 14 days, and by 21 days the PICP-treated tumors had larger vessels than contro-lateral control tumors, these differences persisting up to 48 days (Fig. 2). The vessels detected in PICP-treated tumors since 14 days were pervious and there was an outstanding difference from control tumors in the presence of red cells in these vessels (identified also by tricromic Masson's stain, not shown).

**Figure 3:**
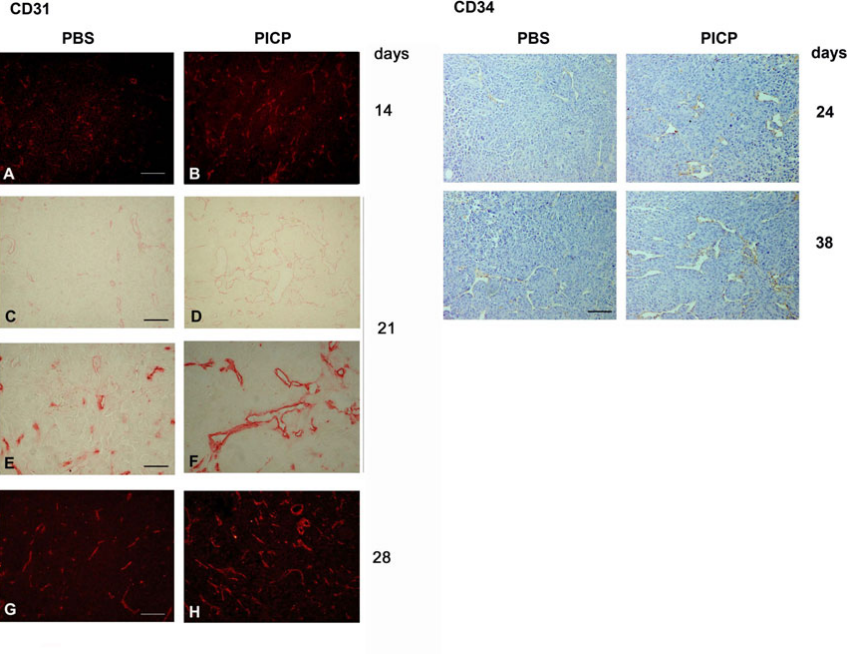
**Vessel formation in subcutaneous tumors in xenografts of MDA MB231 in presence or absence of PICP**. Immunohistochemistry and immunofluorescence identify CD31- and CD34-expressing endothelial cells. Exemplary micrographs of tumors are shown out of 3 to 9 tumors examined for each time point. Left: Sections from criopreserved tumors dissected after 14, 21 and 28 days are processed for immunohistochemistry with CD31 antibody, followed by a secondary antibody conjugated either with TRITC (A, B and G, H) or biotin (C-F). Bar is 50 μm for A-D and G-H, and 25 μm for E-F. Sections from paraffin embedded tumors dissected after 24 and 38 days are decorated with antibody to CD34 and revealed by Immunoperoxidase.100× Bar is 50 μm.

Endothelial cells in the vessels were stained with an antibody to CD31 in slices from frozen tumors, or with an antibody to CD34 in paraffin embedded tumors. These antibodies detected endothelial cells at earlier times and with higher frequency in the PICP-treated tumors (Fig. 3, left panel) and confirmed the persistence in time of a more developed vascularization in PICP treated tumors (Fig. 3, left and right panels). Given the ramification and thus, presumably, the tridimensional extension of the vessels found in PICP-treated tumors we decided not to present the data as vessel count/per area, which would not add substantial information on and might mis-represent their numbers and we show only the evidence obtained by the histology and by immunodetection studies. The effect of PICP in promoting rapid vessels formation and organization might be resultant from its chemoattractant effect on endothelial cells, shown in vitro studies [37].

**Figure 4:**
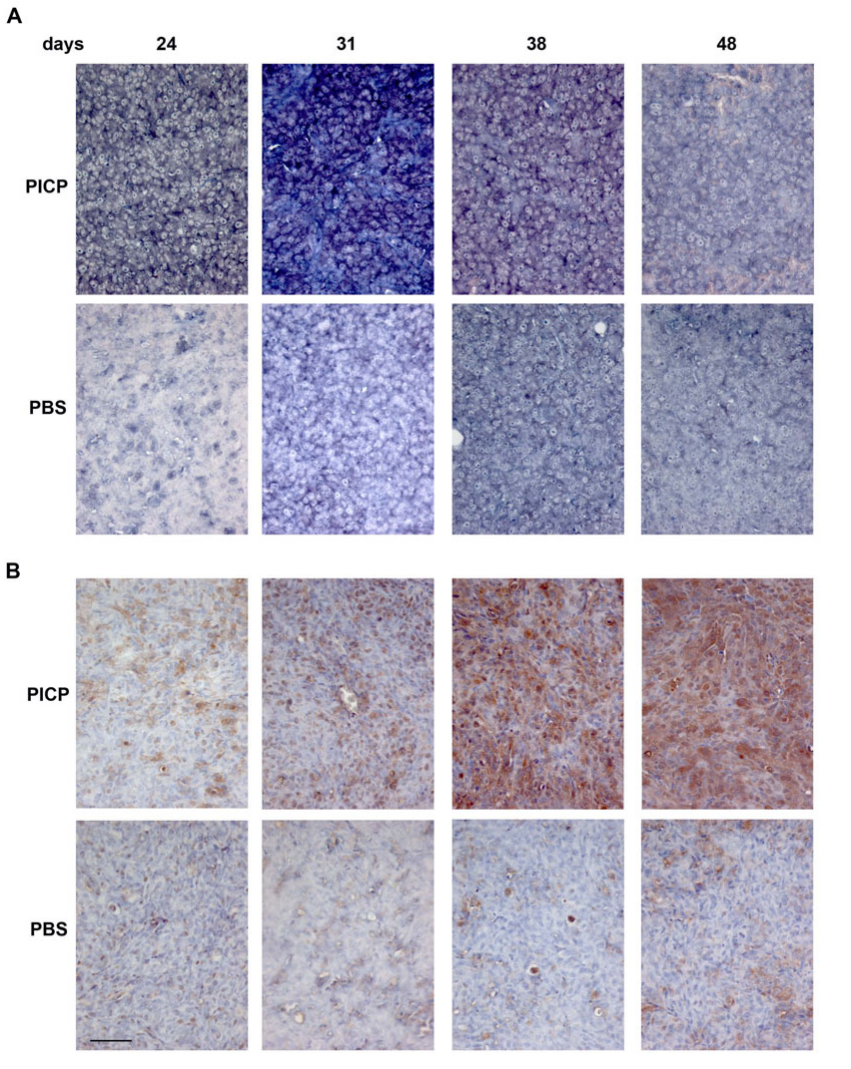
**MMP-9 expression in PICP-treated and untreated contro-lateral tumors**. Micrographs are representative of the results obtained from the analysis of at least two series of contro-lateral tumors treated or untreated with PICP. On the top is indicated the day after implant when the tumors were explanted. The two topmost rows are analysis by ISH and the two lower rows are analysis by immunoperoxidase for MMP-9. Bar is 25 μm.

**Figure 5:**
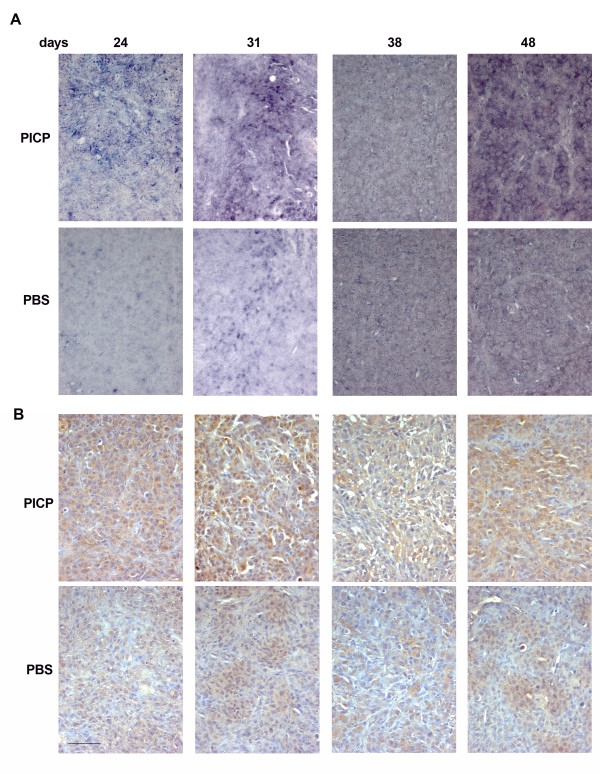
**VEGF-A expression in PICP-treated and untreated contro-lateral tumors**. Micrographs are representative of the results obtained from the analysis of at least two series of contro-lateral tumors treated or untreated with PICP. On the top is indicated the day after implant when the tumors were explanted. The two topmost rows are analysis by ISH and the two lower rows are analysis by immunoperoxidase for VEGF-A. Bar is 25 μm.

**Figure 6:**
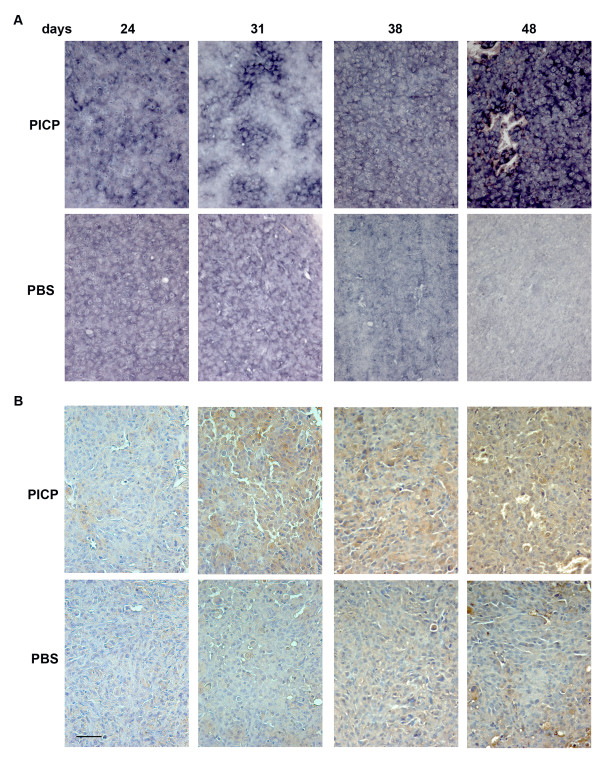
**CXCR4 expression in PICP-treated and untreated contro-lateral tumors**. Micrographs are representative of the results obtained from the analysis of at least two series of contro-lateral tumors treated or untreated with PICP. On the top is indicated the day after implant when the tumors were explanted. The two topmost rows are analysis by ISH and the two lower rows are analysis by immunoperoxidase for CXCR4. Bar is 25 μm.

By In Situ Hybridization we analyzed the tumors for the expression of 3 genes whose co-expression is considered a metastatic signature in breast carcinomas, MMP-9, VEGF-A and CXCR4 (Figures 4 to 6, top two rows). During the development of the tumors the level of mRNA of each gene changed with a slightly different temporal pattern.

For MMP-9 (Fig. 4) the highest mRNA expression was found in PICP-treated tumors at 24 and 31 days, declining thereafter (Fig. 4 top row). This corresponded to a steady increase in number of cells positive for the protein by immunostain up 48 days of growth (Fig. 4, third row) in PICP-treated tumors. The control tumors showed lesser expression of MMP-9 mRNA at all times, localized to individual cells (Fig. 4 second row). Single cells or, only at time 48 days, small clusters of cells were positive for the protein, detected by immunostain (Fig. 4 bottom row).

For VEGF-A (Fig. 5), PICP-treated tumors showed higher expression of mRNA than control tumors at all times of explant (Fig. 5, top 2 rows). In both PICP-treated and control tumors, the highest level of expression of mRNA was at 48 days and the expression was detected in the majority of the tumor cells in PICP-treated tumors. By immunostain of the protein (Fig. 5, bottom two rows), VEGF-A expression was more diffuse in PICP-treated tumors, while in control tumors the positive cells grouped in clusters.

For CXCR4 (Fig. 6) the level of mRNA expression at all times was much higher in the PICP-treated tumors than in contro-lateral tumors (Fig. 6, two top rows). In these first, high expression was confined to large clusters of cells at 24 and 31 days and virtually all the cells expressed CXCR4 mRNA by 48 days. The expression of the protein followed in amounts that of the mRNA, with compartments more intensely stained than others up to 38 days, and virtually all the cells positive for CXCR4 in PICP-treated tumors explanted after 48 days (Fig. 6, third row). Less cells and less intensely stained were seen in the corresponding control tumors (Fig. 6, bottom row).

The treatment with PICP thus caused precocious expression of the above mentioned genes, and supported their increased expression during tumor progression. These data show the induction of a metastatic-prone phenotype by the tumor cells in the presence of PICP. We suggest that this occurs by an epigenetic event, since, by cell growth and death measures and considering the overall tumor growth, there is no significant difference between treated and untreated tumors that could accommodate selective advantage for clones of cells that in presence of PICP would overgrow the other cells, unless these are already at time of implant a majority of the population. In addition, in vitro studies in the MDA MB231 cells used for these implants have shown that the induction of VEGF-A and CXCR4, involving the majority of cells, occur in a timely sequence upon treatment with PICP in a time lapse equivalent to less that 2 population doublings [36].

It is likely that, once the high concentrations of PICP in the implant chemoattracts endothelial cells, the enhanced expression induced by PICP of the 3 gene products described above will determine also the creation of a microenvironment that favors endothelial cells growth and organization in vessels [35-37]. In particular, VEGF-A acts as survival factor for endothelial cells, and its up-modulation by PICP in the tumor can determine paracrine stimulation of endothelial cells. We have previously demonstrated that Nrp-1 induces high levels of VEGF-A expression via p38/PKC mediated pathways, and that up-regulation of CXCR4 could result from the activation of an autocrine loop of VEGF/CXCR4 [36]. In vivo, this could make the tumor cells able to activate the paracrine/autocrine loop CXCR4/CXCL-12/VEGF, when CXCL-12 is expressed in the breast stroma.

**Figure 7:**
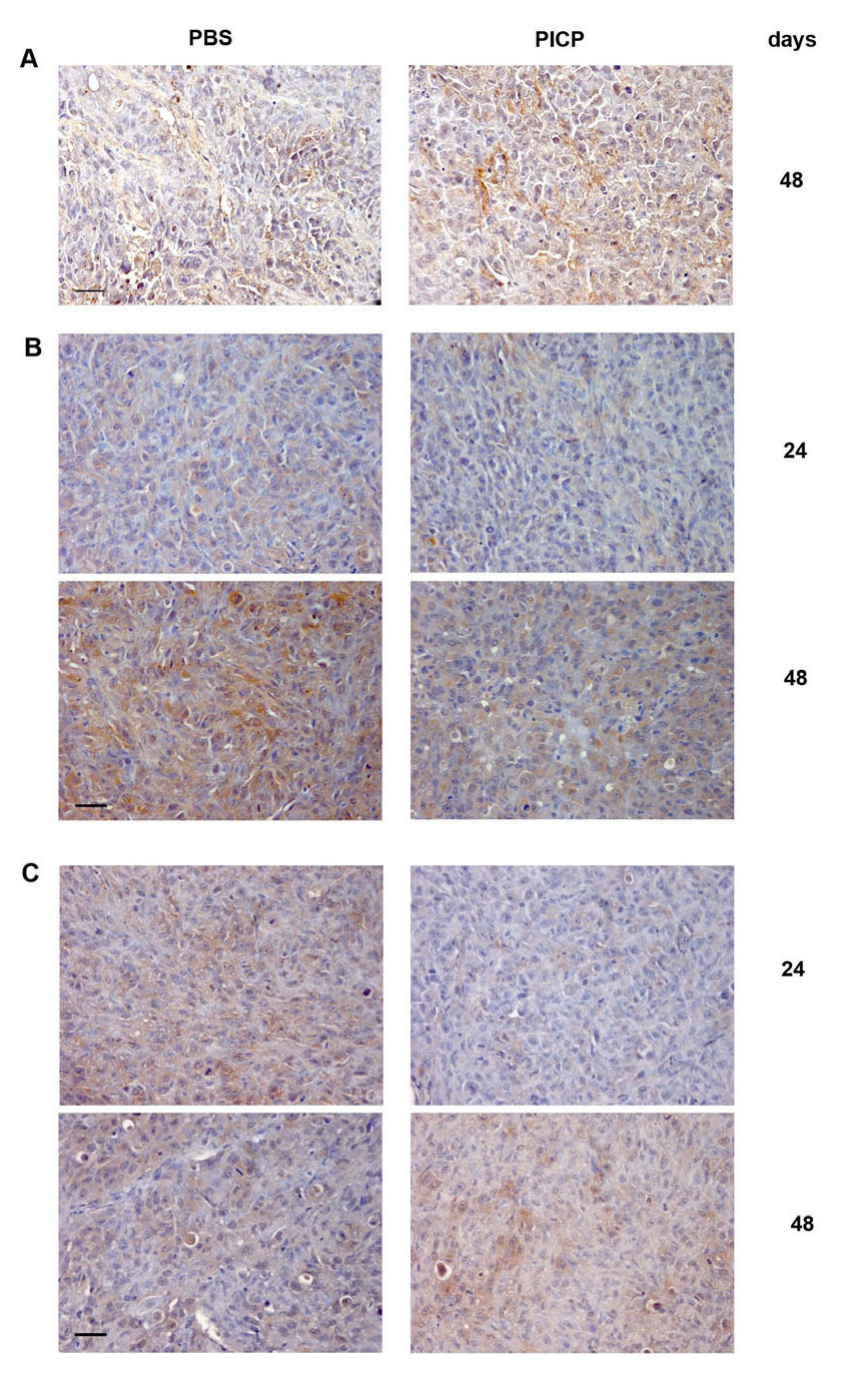
**CXCL-12, RECK and TIMP-2 expression in PICP-treated and untreated contro-lateral tumors**. Micrographs are representative of the results obtained from the analysis by immunoperoxidase of at least two series of contro-lateral tumors treated or untreated with PICP. On the right side is indicated the day after implant when the tumors were explanted. A) with antibody to CXCL-12, B) with antibody for RECK; C) with antibody for TIMP-2. Bar is 25 μm.

We tested the tumors for CXCL-12 expression by immunohistochemistry. In both untreated and PICP-treated tumors CXCL-12 was not detectable up to 38 days, and it was detected in the stromal compartment of both kind of tumors only at day 48, with a slight higher intensity and diffusion in PICP-treated tumors (Fig. 7A). This suggests that the induction of specific genes and the formation of vessels in PICP-treated tumors is a CXCR4/CXCL-12-independent event, that occurs early during tumor development, and possibly preceding the effects on tumor growth and vascularization reported to occur at later times in an in vivo model of co-implant of CAF and tumor cells [14], or reported by implanting an highly metastatic cell line in experiments with timings of explants similar to ours [38].

We have also tested the tumors for other gene products that are known to affect the proteolysis of ECM, which is associated to angiogenesis and metastaticity. We show that the expression of RECK is up modulated in control tumors from 24 to 48 days and much less so in PICP-treated tumors (Fig. 7B). The inhibition of RECK up-regulation mediated by PICP is coherent with the higher level observed for MMP-9, which is directly negatively regulated by RECK. Low expression of RECK is associated to the acquisition of pro-metastatic capability in various tumors [29,30].

The expression of TIMP-2 decreases in time in control tumors, while it increases in PICP-treated tumors (Fig. 7C). Not withstanding the inhibitory role of TIMP-2 on MMPs activity and its MMP-independent inhibitory role in angiogenesis, TIMP-2 is first of all required in stechiometric amounts for MMP-2 activation by MT1-MMP [31]. We have shown MMPs to be induced in vitro by PICP treatment of MDA MB231 cells [36]. TIMP-2 was also shown to have alternative membrane receptors to MT1-MMP, which determine inhibition of endothelial cell proliferation and induce the expression of RECK [41]. However, the fact that we detected a lesser expression of RECK in PICP-treated than in control tumors, suggests that the up-regulation of TIMP-2 observed in the former tumors does not increase RECK expression in our model. It has already been suggested that the prevalent function of TIMP-2, among its multiple potential roles, is dependent from the specific biological context [42] and this concept has accommodated the discrepant data on the modulation of TIMP-2 in different kind of tumors. Given that PICP induces higher level of expression also of MT1-MMP in these xenograft tumors (our unpublished data), we suggest that the increase in TIMP-2 production could be titrated to the membrane in activation complexes with MMPs.

## Conclusion

PICP induces rapidly a metastasis-prone phenotype in the cells of ectopic tumors developed by skin engrafted MDA MB231 cells, not accompanied by effects on their growth. The pro-metastatic changes include increase in the level of expression of MMP-9, VEGF-A, CXCR 4 and decrease in expression of RECK. The joint modulation of the levels of expression of these genes in tumors has a prognostic value for their metastacity. PICP also induces early and sustained development of vessels in the treated tumors. Pro-angiogenic factors are increased in the PICP treated tumor context, where endothelial cells are recruited by chemoattraction from PICP. We envisage that here they find a microenvironment prone for vessel formation and growth.

In PICP-treated tumors the concurrent low expression of RECK and high expression of MMP-9, and the induction in tumor cells expression of MT1-MMP and MMP-2 will push up the balance of proteolysis compared to control. In this context the increase in expression of TIMP-2 might also favor proteolysis. This would support tumor and endothelial cells mobility.

All the changes described occur before detection of CXCL-12 in the tumor stroma. The organization of stromal sects in these implants is a relatively late event, occurring after 31 days. Only when these stromal sects are detected, immunoreactivity for CXCL-12 is associated to the stromal cells and this is not greatly different in control or PICP treated tumor, leading us to suggest that all the effects of PICP we have detected are independent from CXCR4/CXCL-12 signaling.

Overall, the changes induced by PICP in tumors are consistent with the sequential induction of gene expression reported in vitro. They describe its role as a tumor promoter acting early during tumor development and affecting multiple events involved in the phenotypic transition of tumor cells to pro-metastatic cells, as well as favoring the endothelial cells colonization of the implant and the formation of vessels within the tumors. The concomitant occurrence of these changes could facilitate the diffusion of primary tumors, a fact we did not yet investigate.

The pleiotropic effect of PICP on tumor phenotype and its chemoattraction of endothelial cells offer the possibility to investigate means to target the early epigenetic switch it induces in primary tumors, a strategy that might prove more efficient in the containment of tumors, that targeting a single up regulated metastatic gene.

In addition, the response to PICP of MDA MB321 cells might offer a rationale for their preferential homing and the better development of breast cancer metastasis in a PICP-rich environment like bone. It can be hypothesized that PICP could chemoattract the tumor cells preferentially to the bone environment and that it could support their osteolytic activity in this location. These aspects require further investigation.

## Competing interests

The authors declare that they have no competing interests.

## Authors' contributions

DV has prepared the nucleic acid probes and has performed the ISH studies, acquired the images and participated to the drafting of the manuscript and figures; DP has participated to the design of the experiments, prepared PICP, managed cell cultures and the preparation of implants, acquired the images and participated to the drafting of the manuscript and figures; AS has done the immmunohistochemistry experiments; SA has retrieved and measured the explants and done morphology and immunofluorescence decoration of tumors; OB has performed all the animal-related procedures; CG has done the determination of mitotic and apoptotic indexes; AZ has performed preparation of slides; PM conceived of the study, participated to design it, coordinated it and drafted the manuscript. All authors read and approved the final manuscript.

## Pre-publication history

The pre-publication history for this paper can be accessed here:

http://www.biomedcentral.com/1471-2407/9/59/prepub
